# Clinical effects and biological mechanisms of exercise on lumbar disc herniation

**DOI:** 10.3389/fphys.2024.1309663

**Published:** 2024-01-16

**Authors:** Ziwen Wang, Xindai Liu, Ke Gao, Haowen Tuo, Xinxin Zhang, Weiguo Liu

**Affiliations:** ^1^ College of Physical Education and Health, Guangxi Normal University, Guilin, China; ^2^ College of International Culture and Education, Guangxi Normal University, Guilin, China

**Keywords:** exercise, lumbar disc herniation, clinical effect, biological mechanisms, review

## Abstract

Lumbar Disc Herniation (LDH) is a syndrome in which lumbar disc degeneration, rupture of the annulus fibrosus, and herniation of the nucleus pulposus irritate and compress the nerve roots and cauda equina, resulting in the main manifestations of lumbar pain and/or lower extremity pain. There is evidence in various clinical areas that exercise is effective in treating LDH, and exercise intervention for more than 2 weeks reduces disease activity in LDH. However, the mechanism of exercise’s action in reducing disease activity in LDH is unclear. In this article, we first summarize and highlight the effectiveness of exercise in treating LDH and provide guideline recommendations regarding exercise type, intensity, frequency, and duration. Then, we integrate the existing evidence and propose biological mechanisms for the potential effects of exercise on neuromechanical compression, inflammatory chemical stimuli, and autoimmune responses from the perspective of LDH pathogenesis as an entry point. However, a large body of evidence was obtained from non-LDH populations. Future research needs to investigate further the proposed biological mechanisms of exercise in reducing disease activity in LDH populations. This knowledge will contribute to the basic science and strengthen the scientific basis for prescribing exercise therapy for the routine clinical treatment of LDH.

## 1 Introduction

Lumbar Disc Herniation (LDH) is a syndrome in which lumbar disc degeneration, rupture of the annulus fibrosus, and protrusion of the nucleus pulposus irritate and compress the nerve root and cauda equina, resulting in lumbar pain and/or lower limb pain (Zheng et al., 2021). In recent years, the incidence of LDH has been increasing year by year, and the incidence among young people has been rising due to the long-term use of sedentary postures for study and work and the reduction of physical activity (Frino et al., 2006). LDH has caused severe impacts on the daily life and work of patients, and the increasing incidence of LDH has caused an enormous burden on society (Katz, 2006). Therefore, the search for a safe, effective, and generalizable method for preventing and treating LDH is a social problem that needs to be solved by medical practitioners today.

The therapeutic mechanism of LDH is closely related to the recovery of low back muscle function. As the slow muscle fibres of the paravertebral muscles are significantly reduced in patients with low back pain, their role in maintaining trunk posture and body position is weakened, and it is easy to have lumbar back muscle fatigue (Mayer et al., 1985). Prolonged lumbar back muscle fatigue will lead to dysfunction of the tissue structure that maintains the endogenous and exogenous stability of the lumbar spine, thus gradually losing the function of maintaining the spine’s stability, leading to or aggravating LDH (Stevens et al., 2007). Despite the availability of several surgical and non-surgical measures for treating LDH (Loupasis et al., 1999), previous studies have shown that 85% of patients prefer to be treated in non-surgical treatment (Kreiner et al., 2014). This may be because the two treatments have no significant difference in therapeutic efficacy (Yorimitsu et al., 2001). More importantly, non-surgical conservative treatment measures dominated by exercise therapy are highly beneficial in enhancing the function of lower back muscles (McGill, 1998) The 2007 clinical practice guideline “Diagnosis and treatment of lower back pain,” jointly published by the American College of Physicians and the Pain Society, states that there is moderately strong evidence that exercise therapy is effective in the treatment of chronic low back pain (Chou et al., 2007); The United Kingdom National Institute for Health and Care Excellence guideline issued in 2010 added a recommendation for conservative treatment as a first-line treatment and stated that there is strong evidence that exercise therapy is effective in the treatment of chronic low back pain (Bernstein et al., 2017); and continued reports have shown that targeted selection of exercise exercises can enhance low back muscle function and reduce their fatigue (Merritt and Merritt, 2007; Kim et al., 2013; Lee et al., 2013; Gürşen et al., 2016; Sipaviciene and Kliziene, 2020; Li, 2021).

The clinical effects of exercise on LDH have been extensively studied. However, to the best of our knowledge, the biological mechanisms of how exercise promotes recovery from LDH have not yet been fully explored. Exploring the underlying biological mechanisms is essential for understanding the pathogenesis of LDH and proposing new exercise therapies.

## 2 Clinical effects of exercise on patients with LDH

To summarize the evidence for the clinical effects of exercise in LDH, we conducted a literature search on PubMed and Google Scholar using keywords (exercise, physical activity, clinical effects, and lumbar disc herniation) to identify relevant trials and review articles. Because there were overlapping trial and review articles, and some review articles had a specific focus (e.g., a particular type of exercise or clinical area). This article only reviews the 15 randomized controlled trial (RCT) studies published between 2009 and 2023 from the search results. The characteristics of the included articles and the effects of the exercise intervention are shown in Table 1.

**TABLE 1:** Characteristics of included studies.

Study		LDH patient	Exercise						Clinical effects					
Author, year	Design	N, age	Intervention group (IG)			Control group (CG)			JOA	ODI	VAS	ROM	NHP or QOL	Effectiveness of the intervention
			Type	Frequency and Duration	Time	Type	Frequency and Duration	Time						
Taşpınar G, et al. (Taşpınar et al., 2023)	RCT	54, 50.3 ± 6.7	Pilates exercise therapy	6 weeks × 3 sessions	45–60 min	Routines without doing any exercises	6 weeks × 3 sessions × 30 min	45–60 min		↑	↑		↑	**After 6 weeks of exercise** Joint pain (VAS), functional disability (ODI), and degree of improvement in mood depression (QOL) were significantly improved. (IG vs CG)
Selim M N, et al. (Selim et al., 2022)	RCT	15, 48.5 ± 5.8	A.Mulligan spinal mobilization with leg movement and transcutaneous electrical nerve stimulation B. McKenzie and transcutaneous electrical nerve stimulation	4 weeks × 3 sessions	NR	Transcutaneous electrical nerve stimulation	4 weeks × 3 sessions ×	30 min		↑	↑			**After 4 weeks of exercise** Pain conditions (VAS) and functional status (ODI) were significantly improved. (IG vs CG)
Deniz Bayraktar et al. (Bayraktar et al., 2016)	RCT	31, 41.5 ± 23.5	Water specific therapy	8 weeks × 3 sessions	60 min	Bridging, trunk-curl, quadrupedal, side lying, sitting on a ball and standing	8 weeks × 3 sessions	60 min		—	—		—	After 4 weeks of exercise Pain conditions (VAS), functional status (ODI) and degree of improvement in mood depression (NHP) had no significant effect
Shen zhixiang, et al. (Shen et al., 2009)	RCT	30, 47.2 ± 11.7	Swiss ball exercises and lumbar traction	4 weeks × 6 sessions	30 min	Lumbar traction	NR	NR			↑			**After 4 weeks of exercise** Joint pain (VAS) were significantly improved. (IG vs CG)
Yildirim P, et al. (Yildirim and Gultekin., 2022)	RCT	48, 37.9 ± 7.5	Yoga exercise and patient education	12 weeks × 2 sessions	60 min	Patient education and routines without doing any exercises	12 weeks × 2 sessions	60 min		↑	↑			**After 12 weeks of exercise** Pain conditions (VAS), and functional status (ODI) were significantly improved. (IG vs CG)
Iosub, Monica Elena, et al. (Iosub et al., 2023)	RCT	77, 50. ± 13.1	Vojta therapy and procedures, mobility, strength exercises and motor control exercise	2 weeks × 5 sessions	80 min	Mobility and strength exercises and motor control exercises	2 weeks × 5 sessions	50 min		↑	↑		↑	**After 2 weeks of exercise** Pain conditions (VAS), disability level, mobility (ODI), strength, and health-related quality of life (NHP) were significantly improved. (IG)
Gulsen, Mustafa, al. (Gulsen et al., 2019)	RCT	64, 53.0 ± 14.6	A.Lumbar stabilization training B.proprioceptive neuromuscular facilitation C.physical therapy	4 weeks × 5 sessions	45 min	Without any application	NR	NR		↑	↑			**After 2 weeks of exercise** Muscle strength and endurance in the lumbar, pain conditions (VAS), and functional status (ODI) improved significantly. (IG vs. CG)
Xu, J. et al. (Xu et al., 2020)	RCT	72, NR	Shi-style spine balance manipulation combined with Daoyin therapy	4 weeks × 3 sessions	20 min	Lumbar mechanical traction	4 weeks × 3 sessions	20 min		↑	↑			**After 4 weeks of exercise** Pain conditions (VAS) and comfort level (ODI) were significantly improved. (IG vs. CG)
Zhou Xin et al. (Zhou et al., 2022)	RCT	270, 40.0 ± 20.0	Traditional Chinese exercise combined with massage	6 weeks × 3 sessions	30 min	Traditional Chinese massage	6 weeks × 3 sessions	30 min		↑	↑	↑		**After 6 weeks of exercise** Pain conditions (VAS), functional status (ODI), and lumbar spine activities (ROM) were significantly improved. (IG vs CG)
Khanzadeh, R. et al. (Khanzadeh et al., 2020)	RCT	30, 40.3 ± 7.7	Suspension core stability exercises	8 weeks × 3 sessions	60 min	Conventional core stability exercises	8 weeks × 3 sessions	30 min			↑			**After 8 weeks of exercise** Joint pain (VAS) was significantly improved. (IG vs CG)
França F R et al. (França et al., 2013)	RCT	23, 45.1 ± 6.3	Exercises of lumbar segmental stabilization	8 weeks × 2 sessions	60 min	Electrotherapy	8 weeks × 2 sessions ×	60 min		↑	↑			**After 8 weeks of exercise** Joint pain (VAS) and functional status (ODI) were significantly improved. (IG vs CG)
Javaheri A H (Javaheri et al., 2011)	RCT	30, 41.6 ± 5.0	Exercise Therapy and Massage	8 weeks × 3 sessions	60 min	No special activity	8 weeks × 3 sessions	60 min					↑	**After 8 weeks of exercise** Quality of life (QOL) improved significantly. (IG vs CG)
Lu Weiwei et al. (LU et al., 2014)	RCT	50, 43.8 ± 5.6	Physiotherapy core stability exercise and Proprioceptive training	8 weeks × 6 sessions	35 min	Physiotherapy and core stability exercise	8 weeks × 6 sessions	15 min		↑	↑			**After 6 weeks of exercise** Pain conditions (VAS), muscle strength, and comfort level (ODI) were significantly improved. (IG vs CG)
Dae-Keun Jeong et al. (Jeong et al., 2017)	RCT	30, 33.3 ± 9.4	Balance center stabilization resistance exercise	4 weeks × 3 sessions	30 min	three-dimensional stabilization exercise group	4 weeks × 3 sessions	30 min		↑				**After 4 weeks of exercise** Pain conditions and muscle strength (ODI) were significantly improved. (IG)
Liu Ming et al. (Liu and Gao., 2020)	RCT	120, 38.72 ± 2.37	Conventional traction therapy and Mulligan technique and Swiss ball exercises	2 weeks × 7 sessions	60 min	Conventional traction therapy	2 weeks × 7 sessions	30 min	↑		↑			**After 2 weeks of exercise** Pain conditions (VAS), Restoration of functionality (JOA), and living conditions were significantly improved. (IG vs CG)

Notes: RCT, randomized controlled trial; JOA, japanese orthopedic association; ODI, oswestry disability index; VAS, visual analog scales; NHP, nottingham health profile; QOL, quality of life; ROM, range of motion; ↑ Indicate clinical effects improved; — Indicate no significant effect; NR, not reported. Bold values represents the duration of the intervention.

Exercise has a multifaceted positive impact on the clinical outcome of LDH. Firstly, exercise could effectively reduce pain (Shen et al., 2009; França et al., 2013; Gulsen et al., 2019; Khanzadeh et al., 2020; Xu et al., 2020; Selim et al., 2022; Yildirim and Gultekin, 2022; Zhou et al., 2022; Iosub et al., 2023; Taşpınar et al., 2023), improve lumbar spine motion limitation (Zhou et al., 2022; Iosub et al., 2023), and significantly increase lumbar spine range of motion during forward flexion and backward extension. Secondly, exercise could be effective in improving quality of life, mental health, or sleep status (Javaheri et al., 2011; Taşpınar et al., 2023) so that patients could maintain a positive and sunny attitude towards the disease without exacerbating the severity of the disease activity or particular symptoms. In addition, some specific exercises could enhance muscle coordination, flexibility, and balance, thus improving the stability of the lumbar spine (França et al., 2013; LU et al., 2014; Gulsen et al., 2019). In sum, exercise interventions positively affect rehabilitation and overall health in patients with LDH.

As shown in Table 1, the effectiveness of exercise interventions may be related to types, duration, intensity, and frequency. Regarding exercise types, this study found that the included studies were all based on non-acute self-weighted exercises for the lumbar and back core muscles and that different types of exercises had different effects on clinical effectiveness. Specifically, Mulligan spinal mobilization with leg movement was more effective than the McKenzie method (Selim et al., 2022), lumbar stabilization training was more effective than proprioceptive neuromuscular facilitation (LU et al., 2014), and suspension core stability training was more effective than traditional core training (Khanzadeh et al., 2020). However, current research lacks comparative studies of the effects of multiple exercise-type interventions, so the optimal type of exercise is unclear. Regarding exercise duration, previous studies have shown that exercise training for 2–12 weeks or longer significantly improves physical performance and reduces the clinical severity of disease in patients with LDH (Yildirim and Gultekin, 2022). However, a 1-week exercise intervention was ineffective in patients’ disease recovery (Li et al., 2022). Therefore, concerning the results of the current studies, exercise interventions in patients with LDH should be at least 2 weeks to enhance physical performance and reduce disease severity significantly. Regarding exercise intensity, the included studies did not finely classify exercise intensity. However, previous studies have shown that high-intensity strength training (1RM ≥ 70%) and aerobic training (maximal heart rate or maximal oxygen consumption ≥70%) have a negative effect on some elderly patients with LDH, producing symptoms such as joint damage (Saal, 1996). Therefore, the intensity of training should be rationally arranged according to the patient’s condition when providing exercise interventions for patients, and large-intensity exercise interventions should be avoided as much as possible. In terms of exercise frequency, Kim et al. (Kim et al., 2010) found that the follow-up exercise intervention in 40 patients with LDH found that the patients two times/a week had significantly increased their lumbar strength, decreased their Oswestry dysfunction index and significantly decreased their back pain and leg pain scores. The patients who had one time/2 weeks and those who did not train had significantly decreased their lumbar strength, suggesting that the frequency of exercise should be kept at least two times/a week in order to have a significant effect.

In conclusion, exercise could improve physical performance, reduce pain, improve quality of life, improve mental health and sleep, and relieve lumbar fatigue in patients with LDH. However, it is necessary to investigate further what types of exercise, as well as the duration, frequency, and intensity of exercise, are most effective in treating LDH in the future.

## 3 Biological mechanisms of exercise in the treatment of LDH

**FIGURE 1:**
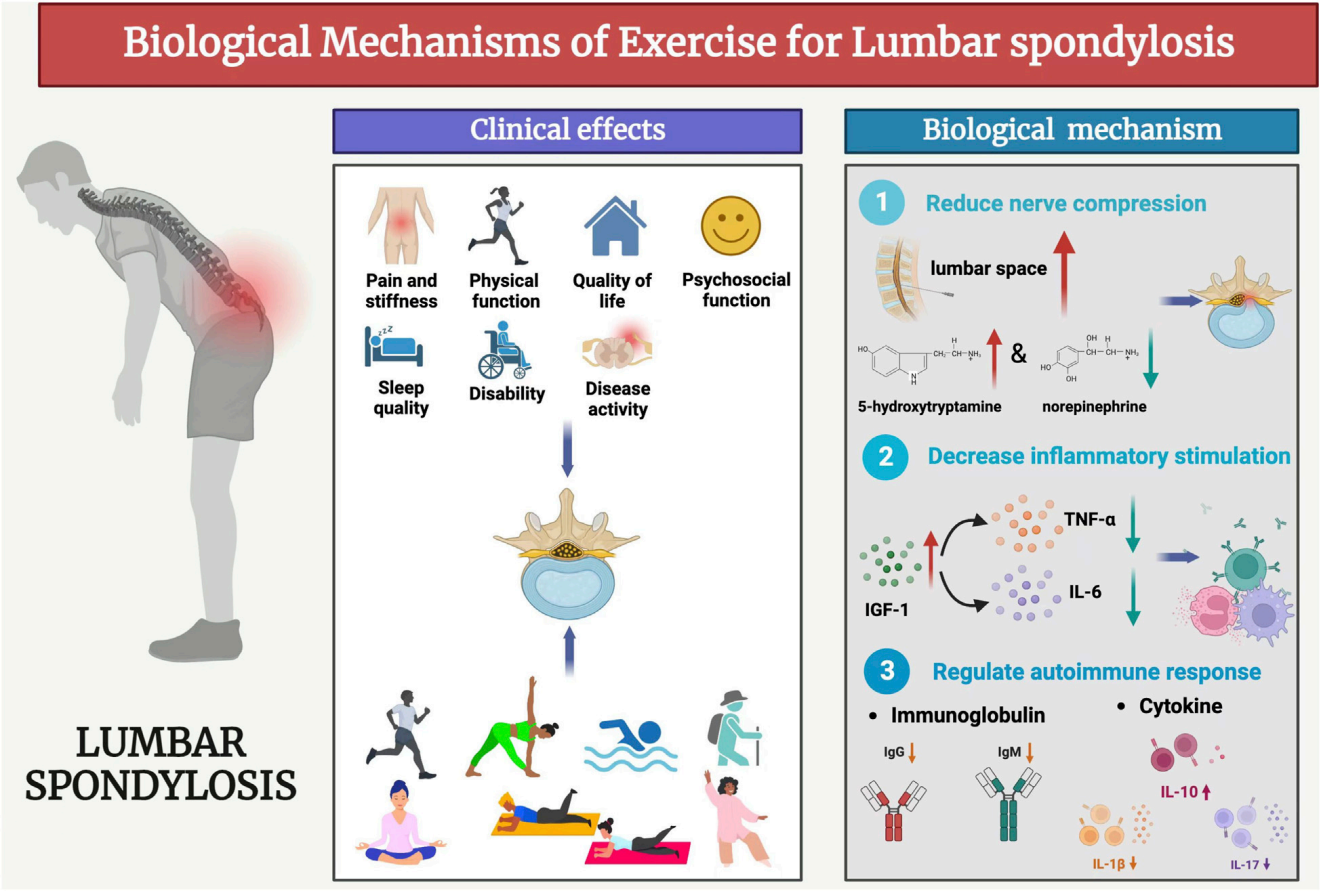
Biological mechanisms of exercise for lumbar spondylosis.

In recent years, the pathogenesis, diagnosis and treatment of LDH have been gradually improved through many experimental and clinical studies. Previous studies have concluded that the biological mechanisms of LDH pathogenesis are the mechanical compression doctrine, inflammatory chemical stimulation doctrine, and autoimmune doctrine (Meng et al., 2022). Exercise could produce a series of biological responses to the three doctrines and promote the recovery of LDH (Figure 1).

### 3.1 Biological mechanisms of exercise to reduce mechanical compression of nerves

The previous study found that taijiquan exercise positively affected LDH (Deng and Xia, 2018), and both peroneal motor and sensory nerves were significantly improved (Zou et al., 2019). This suggests that tai chi exercise improves the stability of the lumbosacral spine, relieves the compressed lumbosacral nerves to varying degrees, and then improves the conduction function of the peripheral nerves. In addition, previous studies have also shown that exercise significantly increased the metabolic level of the central nervous system mediator 5-hydroxytryptamine (Wipfli et al., 2011) while decreasing the sympathetic mediator norepinephrine in the vegetative nerves (Jia et al., 2020). This means that the central and vegetative functions are adjusted. Interestingly, the results of previous studies also found that herniated discs often do not directly compress the nerve roots but rather cause compression and congestion of the vertebral veins below the intervertebral foramina, limiting reflux, followed by impaired capillary blood flow and finally affecting arterial blood supply (Rydevik et al., 1984; Hoyland et al., 1989). Therefore, vertebral venous stasis is important in radicular pain (Parke and Watanabe, 1985). In this regard, long-term systematic exercise can produce well-adapted changes in human blood viscosity by appropriately decreasing it within a certain range (Pichon et al., 2016).

Based on the above studies, exercise could improve the function of the peripheral tissues of the spine, increase the lumbar spinal space, reduce and release the extrusion of the protruding material on the intervertebral foraminal nerve roots and vertebral organs, prompt the intervertebral disc space to generate negative pressure and reduce the pressure within the lumbar spinal interspace, and effectively improve the microcirculation of the lesion blocking localization (Lam et al., 2018). This may be an important biological mechanism for exercise to reduce the mechanical compression of nerves. However, there are no studies to investigate the effect of exercise on vertebral vein blood viscosity and other blood characteristics in LDH currently, and further research is needed.

### 3.2 Biological mechanisms by which exercise ameliorates inflammatory chemical stimuli

The innate immune system triggers inflammation once immune cells detect infection or tissue damage (Weyand and Goronzy, 2021). Previous studies have pointed out that inflammatory mediators and related cytokines are essential in LDH (Djuric et al., 2019), with tumour necrosis factor-alpha (TNF-α) being a key mediator of the inflammatory response (Driscoll et al., 1997). In addition, Pelosi et al. (Pelosi et al., 2007) found that localized expression of IGF-1 in skeletal muscle by transgenic techniques significantly downregulated the expression of the inflammatory factor TNF-α. The study of Wang et al. further demonstrated that the serum levels of inflammatory factors (such as IL-6 and TNF-α) were negatively correlated with the levels of IGF-1 (Wang et al., 2019). Therefore, IGF-1 has an inhibitory effect on inflammatory factors such as TNF-α and IL-6.

On the other hand, previous studies on exercise interventions have shown that exercise could affect IGF-1 levels in skeletal muscle and the circulatory system (Kim et al., 2019; Norling et al., 2020). IGFBP and IGF-1 levels in skeletal muscle were significantly upregulated in humans after high-intensity aerobic exercise (Kraemer et al., 2017). Resistance training also raises circulation IGF-1 levels (Rojas Vega et al., 2010), with intermittent aerobic exercise being more effective than continuous aerobic exercise (Żebrowska et al., 2018). The above studies have amply demonstrated the facilitating effect of exercise on IGF-1 synthesis, which may be an important biological mechanism by which exercise ameliorates inflammatory chemical stimuli. However, the dependence of IGF-1 on the type, duration, intensity, and frequency of exercise is not clear, so it is of great research value to explore the correlation in the future.

### 3.3 Biological mechanisms by which exercise modulates the autoimmune response

Few studies have investigated how exercise improves LDH by modulating the immune system. However, the mechanisms of the immune system’s action on LDH have been discussed in various studies, mainly focusing on cytokine and immunoglobulins (Naylor et al., 1975; Miyamoto et al., 2000; Shamji et al., 2010). Regarding immune factors, previous studies (Miyamoto et al., 2000; Shamji et al., 2010; Al-Obaidi and Mahmoud, 2014; Djuric et al., 2020) detected the presence of large amounts of IL-1β, IL-17, and IL-10 in the intervertebral discs of patients with LDH and investigated the mechanism of their action on LDH. The results showed a significant negative correlation between the concentrations of IL-1β and IL-17 and the condition of LDH (Al-Obaidi and Mahmoud, 2014; Tan et al., 2022). At the same time, IL-10, a critical immunosuppressive factor, had a significant positive correlation with the condition of LDH (Uçeyler et al., 2007). Although fewer studies investigate the effects of exercise on modulating cytokine in patients with LDH, many previous studies have been conducted in normal populations. Faelli et al. (Faelli et al., 2020) found that IL-1β levels were significantly reduced after 24 sessions of HIFT training. Hoffman-Goetz et al. (Hoffman-Goetz et al., 2010) found that prolonged moderate to moderate-intensity exercise increased IL-10 secretion. In a study by Rahimi et al. (Rahimi and Hormones, 2019), 8 weeks of resistance training reduces IL-17 levels. Regular exercise (Alizadeh et al., 2015; Conroy et al., 2016; Karstoft and Pedersen, 2016) reduces IL-1β, IL-17 and increases IL-10 levels. Interestingly, some studies pointed out that the effect of exercise on cytokine may be related to the intensity and type of exercise, as Peake et al. (Peake et al., 2005) found that, after athletes ran at different exercise intensities, IL-10 was significantly increased in the high-intensity group, while there was no change in the other groups. IL-17 levels increased after high-intensity running but decreased after free exercise (Duzova et al., 2009; Cook et al., 2013). Based on the above findings, the present study concluded that exercise may improve LDH disease by decreasing the concentrations of IL-1β and IL-6 and increasing the concentration of IL-10. However, the improvement effect may be limited by the intensity or type of exercise, which could be further explored in future studies.

Regarding immunoglobulins, previous studies (Naylor et al., 1975; Kang et al., 1996; Duzova et al., 2009) demonstrated that the levels of IgG and IgM were significantly elevated in patients with LDH, and the concentrations of IgG and IgM were positively correlated with the severity of LDH. As with cytokine, few previous studies have investigated the effects of exercise on modulating immunoglobulins in patients with LDH, mainly focusing on studies in normal populations. Previous studies have shown that prolonged high-intensity exercise training reduces IgG, IgM, and IgA concentrations and increases the degree of reduction with increasing exercise load (Coppola et al., 2005). Interestingly, however, a study by Mitchell et al. (Mitchell et al., 1996) showed no significant change in IgG and IgM concentrations after 12 weeks of moderate-intensity exercise. In contrast, Nieman et al. (Nieman and Pedersen, 1999) found a significant increase in IgM and IgG concentrations after prolonged moderate-intensity exercise. This could be a difference caused by the different types of exercise in the two studies. To address the controversial phenomena in the existing studies, future studies should use randomized controlled trials to investigate the effects of different types or intensities of exercise on the immunoglobulins of patients with LDH and also to investigate further how other immunologically active substances act on patients with LDH, in order to find the optimal exercise therapy.

## 4 Conclusion

Extensive research has focused on the clinical efficacy of exercise in treating LDH. Substantial evidence indicates that exercise therapy’s varying types, durations, and intensities are clinically effective for LDH, and in particular, that the use of non-acute self-weighted exercise types, exercise durations exceeding 2 weeks, and non-high-intensity exercise therapies could alleviate disease activity. Nevertheless, most current literature primarily emphasizes clinical observation. It relies on subjective scoring criteria like VAS and JOA in its assessment, lacking quantitative and precise observation indexes to improve its credibility. Consequently, this review begins by examining the pathogenesis of LDH. It proceeds to delve into three facets of the biological mechanisms influenced by exercise: mechanical compression, inflammatory chemical stimulation, and autoimmunity. Subsequently, this review takes the pathogenesis of LDH as an entry point to discuss the biological mechanisms of exercise in three aspects: mechanical compression, inflammatory chemical stimulation, and autoimmunity. However, the relevant evidence is mainly based on non-LDH patients, and the effect of exercise type on the biological response of LDH patients is not yet clear and needs to be further explored.

## 5 Future directions

In response to the existing studies, we suggest that the biological response to exercise should be explored in patients with LDH, and we recommend a long intervention follow-up study. Future research directions can be explored in the following three areas: 1) The effect of exercise on vertebral venous blood viscosity and exploring adaptive changes; 2) Exercise plays a positive role in IGF-1 production, exploring the dependence of IGF-1 on exercise mode and intensity and the inhibitory effect of IGF-1 on the inflammatory factor THF-α; 3) Depending on the intensity and type of exercise, the effects of exercise on other immunoreactive substances were further investigated to reveal the potential modulatory effects of exercise on the immune system of patients with LDH and to determine the optimal exercise treatment measures.
